# Self-Inflicted Strangulation of Prepuce in a Child

**Published:** 2013-01-01

**Authors:** Fikret Halis, Mehmet Inci, Matthew T Freier, Ahmet Gokce

**Affiliations:** Department of Urology, Korfez State Hospital, Kocaeli, TURKEY; Department of Urology, Mustafa Kemal University, School of Medicine, Hatay, TURKEY; Department of Urology, Tulane University, School of Medicine, New Orleans, LA, USA; Department of Urology, Mustafa Kemal University, School of Medicine, Hatay, TURKEY and Department of Urology, Tulane University, School of Medicine, New Orleans, LA, USA

**Keywords:** Penis, Trauma, Foreskin

## Abstract

Penile trauma is an uncommon but potentially serious injury presenting to pediatric emergency room. Strangulation injuries of the penis can be self-inflicted or occur accidentally. However, self-inflicted strangulation of the foreskin in children is rare. This is a case of 7-year-old uncircumcised boy who himself tied foreskin of prepuce with a cotton thread. The thread was cut and circumcision performed to remove the damaged foreskin.

## INTRODUCTION

Penile trauma is uncommon in pediatric patients. Reported traumatic penile injuries include hair tying around penis, zipper injuries, electrical injuries, animal bites, and blunt and/or penetrating trauma [1]. Penile injuries are classified into 4 types according to the anatomical structures involved: skin, erectile tissue, urethra and complex injuries. Injury to the prepuce involves a specific group of patients [2]. Excluding zipper injuries, there are few reports of foreskin injury. Self-inflicted strangulation of foreskin in children is rarely reported. We present a case of foreskin strangulation by patient himself. 

## CASE REPORT

A 7-year-old uncircumcised boy presented to the emergency room with the complaints of acute painful swelling of the foreskin, difficult urination and pain. The symptoms had been present for 12 hours. He and his parents denied any trauma or abuse. On presentation patient was in obvious pain. There was reddened painful area 2 cm � 1 cm� 1 cm at the prepuce. The examination revealed presence of cotton thread tied on the foreskin. Patient was then taken to the operating room for an examination under anesthesia. Examination revealed dark brownish foreskin caused by a cotton thread tied at its base. The proximal penile shaft was normal (Fig. 1). The thread was cut and circumcision performed to remove the damaged foreskin. He was discharged with instructions for local wound care. Parents were advised to consult psychiatric department where following detailed history and examination no abnormality found.

**Figure:**
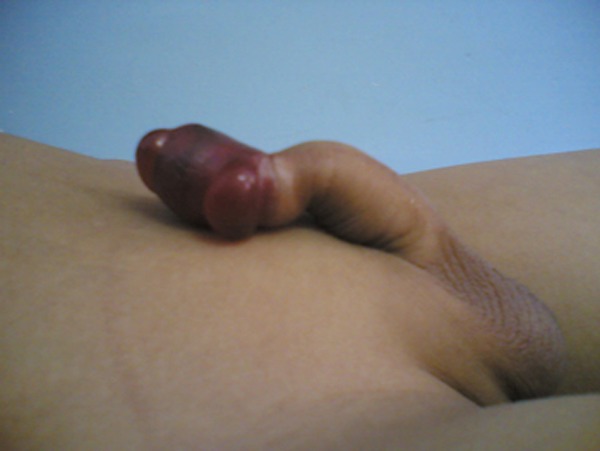
Figure 1: Foreskin strangulation caused by thread.

## DISCUSSION

Penile strangulation injuries range from simple penile engorgement to ulceration, necrosis, urinary fistula, or even gangrene [3]. Strangulation injuries of the penis can occur by self-infliction or accidentally. Self-inflicted strangulation is usually the result of self-mutilation or sexual stimulation; however accidental strangulation may also occur in children [4]. These injuries are graded by Bhat et al as follows [5]:

- Grade I. Edema of distal penis. No evidence of skin ulceration or urethral injury.
- Grade II. Injury to skin and constriction of corpus spongiosum, but no evidence of urethral injury. Distal penile edema with decreased penile sensation.
- Grade III. Injury to skin and urethra but no urethral fistula. Loss of distal penile sensations.
- Grade IV. Complete division of corpus spongiosum leading to urethral fistula and constriction of corpora cavernosa with loss of distal penile sensations.
- Grade V. Gangrene, necrosis, or complete amputation of distal penis.

There are few case reports on injury of the prepuce in children. Zipper injuries of foreskin are relatively frequent in uncircumcised children [6]. Tourniquet injuries occur when bands, rings, or human hair wrap around the penile shaft. Hair thread tourniquet syndrome also called penile tourniquet syndrome has been described in patients of 4 month to 6 year of age [7]. Typically, hair, cotton fiber, or similar material tightly wrapped around the coronal sulcus of almost exclusively circumcised boys causing variable penile injuries ranging from a mild penile edema to penile amputation [8]. It is important to remember that hair tourniquet may be misdiagnosed as balanitis or paraphimosis [9]. Foreskin and simple penile strangulation injury can easily be treated with early intervention. Complications result from delayed recognition and may lead to more serious consequences. Progressive swelling produces arterial insufficiency, leading to ischemia and tissue necrosis [10]. Careful examination of the prepuce in this case led to identification and prompt management of this condition.

## Footnotes

**Source of Support:** Nil

**Conflict of Interest:** None declared
